# Thermochemical and rheological characterization of highly reactive thermoset resins for liquid moulding

**DOI:** 10.1177/00219983231181640

**Published:** 2023-06-07

**Authors:** Leonardo Barcenas, Sidharth Sarojini Narayana, Loleï Khoun, Paul Trudeau, Pascal Hubert

**Affiliations:** 1 Department of Mechanical Engineering, 5620 McGill University, Montreal, QC, Canada; 2 CREPEC - Research Center for High Performance Polymer and Composite Systems, Montreal, QC, Canada; 3 National Research Council Canada, Boucherville, QC, Canada

**Keywords:** Fast curing resin, cure kinetics, rheological properties, liquid injection moulding

## Abstract

Highly reactive thermosets are currently expanding the processability of high-performance structures for transportation industry. The short polymerization time makes it a suitable process to replace metallic structures with polymer matrix-based composite materials. The resin characterization is a fundamental step to obtain the properties and the associated constitutive models, which are required to design and optimize the manufacturing process parameters of composite materials. However, the short time on polymerization requires to use the characterization equipment at their performance capability limits. This work presents a comprehensive methodology to characterize the thermo-chemical properties of highly reactive resin systems, which are relevant for resin impregnation into the preform for liquid injection processes. Four different commercial resin systems are analyzed in this study. Experimental methodologies are analyzed and adapted for best data acquisition at high temperature isothermals. Based on the experimental data, Cure kinetics and viscosity equation-based models are used to describe the behaviour of these complex resin systems. Processing maps are developed based on the cure kinetics and viscosity models to predict the processability time for specific process conditions than can be used on liquid injection moulding processes.

## Introduction

The current global environmental situation requires a greater effort to reduce greenhouse emissions. New challenges for engineers are related to the efficient use of energy and materials. Cheah analyzed the different areas of enhancement to the vehicle design in order to reach these fuel economy targets.^1^ One of these areas is the vehicle weight reduction, which is an important improvement toward higher fuel economy and electric battery range. A lower vehicle mass requires less energy during acceleration due to lower inertial forces. A general rule is that for every 10% reduction in vehicle weight, the fuel consumption of vehicle is reduced by 5%–7 %.^1^

Composites are extensively known as efficient materials due their high specific strength and rigidity. Plastics and polymers composites currently make up about 8% of a vehicle by weight and 50% by volume, and these numbers are expected to increase over time.^2^ The main factors restricting the development of polymer composites in vehicles today are the long production cycle and the cost of fibres.^2^

**Figure 1.**
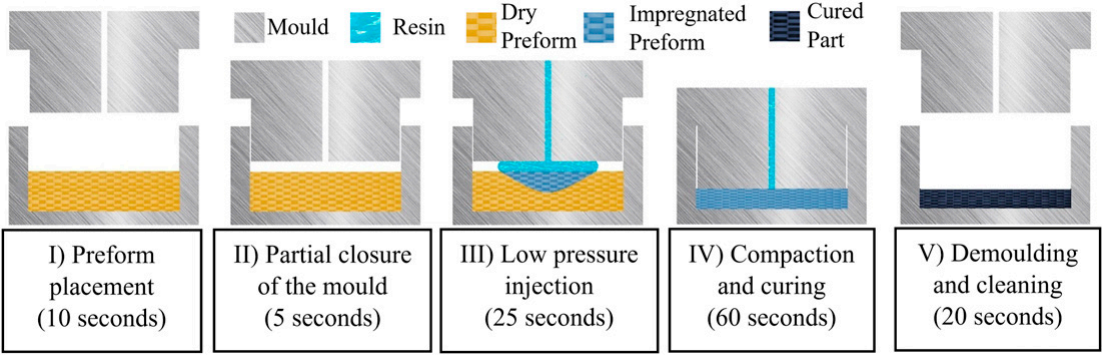
Schematic stages of CRTM process adapted for highly reactive thermosets.

Innovative resin systems require shorter cure cycles (2–5 min) compared with traditional resins (1–2 h).^3^ Highly reactive thermosets are good candidates to shorten the production cycle and accomplish the vehicle production requirement. Manufacturing processes for composite structures in transportation applications should be economic, versatile, and fast. One process with high production volume rate and that is affordable is Compression Resin Transfer Moulding (CRTM). Khoun and Trudeau (2019) describes the implementation of highly reactive thermosets for CRTM.^4^ The process consists of five stages, with an optimal duration of 2 min as seen on Figure 1. The resin is typically injected at room temperature into a pre-heated mould in a range of 100°C–140°C. A strong business case for CRTM was recently demonstrated by NISSAN.^5^

**Figure 2.**
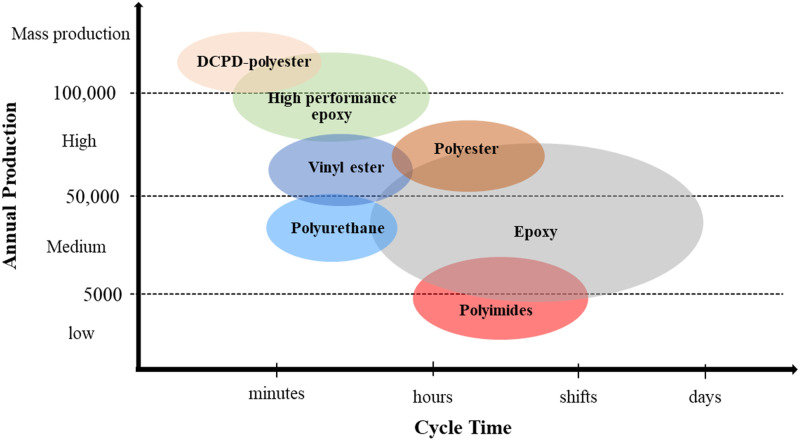
Schematic map of typical resin categories with the time required and their capability for production.

The overall quality of the part made from liquid injection moulding process depends on the impregnation of the preform during the injection. The viscosity of the resin is a fundamental property that drives the impregnation of the preform. The viscosity is strongly dependent to the degree of cure and temperature. As the processing time is considerably shorten, the characterization techniques need to be adapted for the acquisition of the data in a very short time. Figure 2 shows a representative map of some resin categories with the time required for cure.^6–8^ Transportatioin industry requires a high rate production, thus resin systems that cures in seconds to minutes are the most suitable.

A deep comprehensive understanding of the resin processing properties, their constitutive models and the interaction involved is required to define and optimize the parameters of the manufacturing process. Moreover, the high reactivity of the thermosets requires a promptly acquisition of the data from the beginning of the test, which can be challenging because the reaction can occur during the preparation of the sample and setting of the equipment (differential scanning calorimeter and rheometer).

The current characterization techniques are discussed in the methodology section. Improvements on the settings for the differential scanning calorimeter (DSC) and rheometer are discussed to measure the properties of the resin during the highest reaction rates. The implementation of existing material models for the characterization of thermosets has been a source of valuable material data,^9,10^ that is used for further research and process simulation implementation.^11,12^ Different semi-empirial models have been developed to describe the behaviour of cure kinetics^13–18^ and viscosity.^19–22^ The material models used on this manuscript are intended to be simple and accurate. This would facilitate the implementation for process modelling. Four different systems were studied with different techniques and material models from literature.

## Methodology and materials

The key processing properties during the impregnation of the preform is the viscosity and the cure kinetics. A sequence of characterization steps was performed to obtain these properties. First, a thermogravimetric analyser was used to determine the temperature of degradation and the thermal stability of the resin systems. The curing behaviour was then investigated with a differential scanning calorimeter (DSC) to obtain the evolution of the degree of cure, α, as a function of time and temperature. Finally, the viscosity, µ, was measured with a rheometer, and was described as a function of degree of cure, the degree of cure at the gel point, and the temperature.

Based on the high reactivity and processability time, three different commercial thermosets were analyzed (Table 1). One system from Hexion (Epikote 50,475 cured with Epikure 05,500), and two more systems from Gurit: SPX26526 cured with SPX26373 (Gurit Standard) and SPX26526 cured with SPX26180 (Gurit Fast). A slower reactive resin system was analyzed from Olin. This system is the Airstone 780E cured with the 785H hardener, as a reference system. The cure kinetics and viscosity characterization was divided over two research facilities equipped with different equipment suppliers as follows: the National Research Council Canada tested the Hexion and Airstone system while the McGill Structures and Composite Materials Laboratory tested the Gurit Standard and Fast systems. This approach allows for a comparative analysis of the performance and results obtained from each equipment, aiding the identification of their strengths and limitations.

**Table 1.** Specification of the thermoset resins investigated.

Resin		Hardener	Mix ratio. Parts per weight
Hexion	Epikote 50475	Epikure 05500	100:17
Airstone	780E	785H	100:31
Gurit standard	Prime 130 SPX26528	Standard SPX26373	100:25
Gurit fast	Prime 130 SPX26528	Fast SPX26180	100:27

### Thermal stability

**Figure 3.**
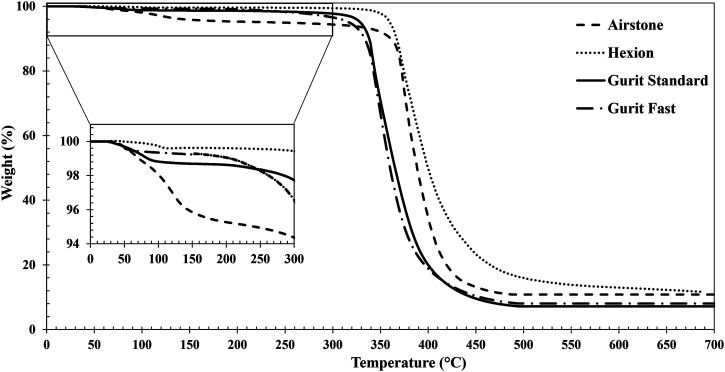
Thermal gravimetric analysis results for the resin systems.

The thermal stability tests were carried out on a thermal gravimetric analyser Q500 from TA Instruments. A temperature ramp at 20°C/min from room temperature to 700°C was applied under air condition. A weight percentage reduction of 5% is observed on Airstone system on the range of the processing temperature of 100°C–140°C as seen on Figure 3. A weight percentage reduction of less than 2% is observed for the rest of the systems over the same temperature range. This is a typical behaviour of thermosets caused by the evaporation of volatiles into the resin as well as any presence of water moisture. The degradation temperature in all the systems is higher than 300°C, and further characterization experiments should be performed below this temperature.

### Cure kinetics

Two different equipment were used with different methodology for the resin systems. The first equipment was a modulated differential scanning calorimeter (MDSC) Q100 from TA Instruments for the Hexion and Airstone resin systems. Dynamic scans were performed at a heating rate of 10°C/min, from −50°C to 250°C. Isothermal scans were performed at 80°C, 90°C, 100°C, and 110°C for both systems. The second equipment was a double-furnace differential scanning calorimeter (DSC) 8500 from Perkin Elmer that was used for the Gurit Standard and Fast resin systems. Dynamic scans were performed for these resins under the same conditions of a heating rate of 10°C/min, from −50°C to 250°C. Isothermal scans were performed at 70°C, 80°C, and 90°C for Gurit Standard. Gurit Fast was tested with isothermals scans of 50°C, 60°C, 70°C, and 80°C.

Highly reactive thermosets present a challenge when characterizing at high isothermal temperatures. The first methodology was implemented on the Q100 DSC, which consist of placing the pan with the uncured resin into the DSC cell at a desired initial temperature. The cell is opened, the sample pan is placed in the cell, then the cell is closed, and the DSC starts acquiring the data. However, the reaction starts while placing the pan and closing the cell. Initial reaction is not monitored, and the data acquire is not fully reliable. The data lost rises as the temperature is higher.

The second methodology was implemented on the Perkin Elmer DSC, where the pan is placed at room temperature. Then the cell is closed, and no reaction is expected during this process. The cell is then heated at the maximum rate of the DSC or 300°C/min. In this method the data is recorded at the beginning of the ramp. In this case there is not lost on data, but the data acquired during the ramp to reach the isothermal temperature is not properly measured. Due to rapid temperature fluctuations, there is a possibility of a time lag or thermal inertia causing a disparity between the furnace temperature and the sample temperature. This leads to uncertainty and errors that increase as the temperature is higher.

### Rheological behaviour

The resin rheological behaviour was analyzed using two types of rheometer. The Hexion and the Airstone resin systems were characterized using AR2000 rheometer from TA instruments. The Anton Paar CTD 600 was used to characterize the Gurit standard and fast resin systems. Both machines used an environmental test chamber and the complex viscosity of the resin was measured in both dynamic and isothermal conditions. Table 2 shows the experimental test matrix used for rheology test and each test was repeated three times for reproducibility. A volume of 0.5–1 ml was placed between two 25 mm parallel plates and trimmed to a thickness between 0.5–1 mm. Strain sweep experiments were conducted to determine the linear viscoelastic range (LVR). For practical reasons, all the tests were conducted at a small oscillatory amplitude of 1 Hz^19^ and the strains used are shown in Table 2.

**Table 2.** Test matrix for rheology tests on highly reactive thermoset resins.

Material resin	Dynamic scan ramp rates (°C/min)	Isothermal scans emperature (°C)	% strain
Hexion	5	80, 100, 110	1.0
Airstone	5	70, 100, 120	1.0
Gurit standard	1,2,5	70,80,90	0.1
Gurit fast	1,2,5	50,60	0.1

#### Dynamic strain control for fast curing resin

The resin systems studied in this work reach minimum viscosity very fast followed by a steep rise is viscosity which is typical of a fast curing epoxy resin.^20^ Large oscillation strains are required at the start of the tests to obtain good signals at low viscosity. However, with the cure progression of the resin, there is an increase in the storage (*G′*) and the loss moduli (*G″*). With this increase, only a small oscillation strain should be used to maintain torque within the capabilities of the instrument. Therefore, to obtain a noise free data over the course of the test, a good oscillation strain control is necessary.

**Figure 4.**
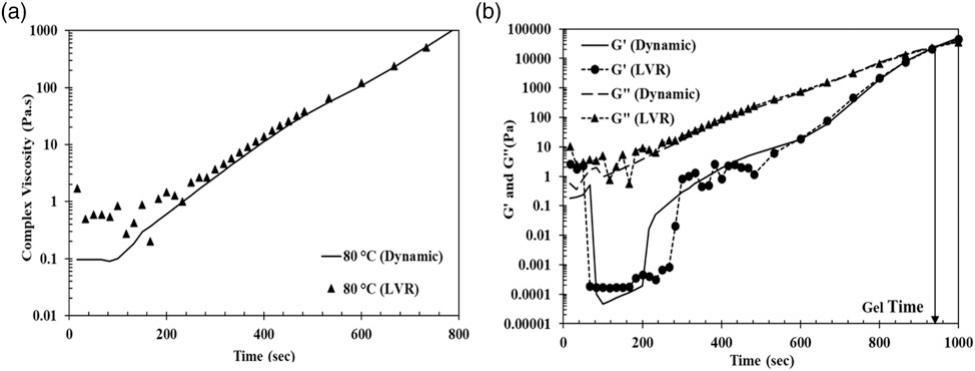
(a) Comparison between Auto-strain (LVR) versus the dynamically controlled strain method for Gurit standard resin system at 80°C and (b) *G′* and *G″* for both tests.

TA instruments uses a method called “Auto-strain” which controls the oscillation strain throughout the cure of the sample.^19^ This technique advices user not to exceed strain beyond LVR. This is ideal for slow and medium cure resin systems. But the resins tested here cure extremely fast and the properties of the resin (*G′* and *G″*) evolve rapidly. As a result, this technique produces noisy data at low viscosity. However, if we use high strains (beyond LVR) at initial stages until the viscosity starts increasing and reduce the strain as the time progresses to get within the LVR range, we can eliminate or minimize noisy data and get a fine curve. Figure 4 shows the comparison of the result using Auto-strain method and dynamically controlled strain method on the Gurit standard resin. The results appear to agree with each other and the high noise in the initial part of the test is eliminated. There was also a match in the *G′, G″* and the gel time data for both tests.

## Cure kinetics modelling

**Figure 5.**
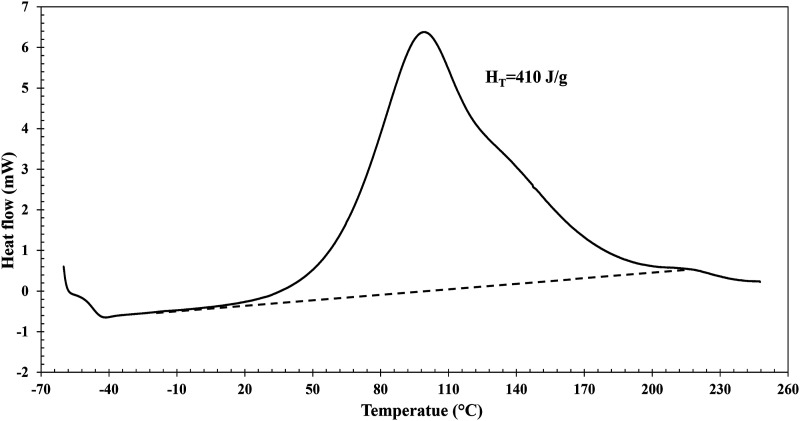
Heat flow of a scanning calorimetry dynamic test at 10°C/min for the Gurit Standard resin system. The dotted line represents the baseline used to calculate the total heat flow of the curing reaction.

The results from the dynamic and isothermal tests are converted into cure rate as the rate of the reaction, dα/dt , is proportional to the heat flow, dH/dt , as seen on equation (1)

(1) $$\frac{d\alpha }{dt}=\frac{1}{H_{T}}\frac{dH}{dt}$$

where *H*_*t*_ is the total specific heat of reaction of the resin. Figure 5 shows the heat flow evolution with time for the Gurit Standard system. The resin total specific heat of reaction is obtained from the area under the curve normalized by the sample mass. For this system, the average H_T_ is 410 ± 3 J/g for a replicated experiment.

The degree of cure, α , is obtained by integrating the area under the curve of the cure rate versus time as seen on equation (2).

(2) $$\alpha =\frac{1}{H_{T}}\int_{0}^{t}\left(\frac{dH}{dt}\right)dt$$

The cure rate can be expressed as a function of the degree of cure into an autocatalytic semi-empirical cure kinetic model. The resin system can be assumed to be mono-reactive considering only one reaction, equation (3),^13,14^ or bi-reactive, where two reactions are assumed to occur, equation (4).^15,16^ Additionally, a diffusion term f(α) can be added to account for low-speed reaction driven by diffusion phenomena, equation (6).^17,18^ Where $E_{ai}$ is the activation energyin J/mol, R is the molar gas constant equal to 8.314 J/K.mol, T is the temperature in K, and $A_{i},m,n$ are fitting constants. $K_{e}$ is the experimental values of $\frac{d\alpha }{dt}$ , and $K_{c}$ is the value of $\frac{d\alpha }{dt}$ from the cure kinetics model with non-diffusion-controlled cure kinetics reaction, where f(α) is equal to 1 for Equations (3) and (4). C , $\alpha_{C0}$ , and $\alpha_{CT}$ are fitting constants for the diffusion term.

(3) $$\frac{d\alpha }{dt}=K_{1}\alpha^{m}{\left(1-\alpha \right)}^{n}f\left(\alpha \right)$$

(4) $$\frac{d\alpha }{dt}=\left({K_{1}+K}_{2}\alpha^{m}\right){\left(1-\alpha \right)}^{n}f\left(\alpha \right)$$

(5) $$K_{i}=A_{i}\exp\left(\frac{-E_{ai}}{RT}\right)$$

(6) $$f\left(a\right)=\frac{K_{e}}{K_{c}}=\frac{1}{1+\exp\left[C\left(\alpha -\left(\alpha_{C0}+\alpha_{CT}T\right)\right)\right]}$$

**Figure 6.**
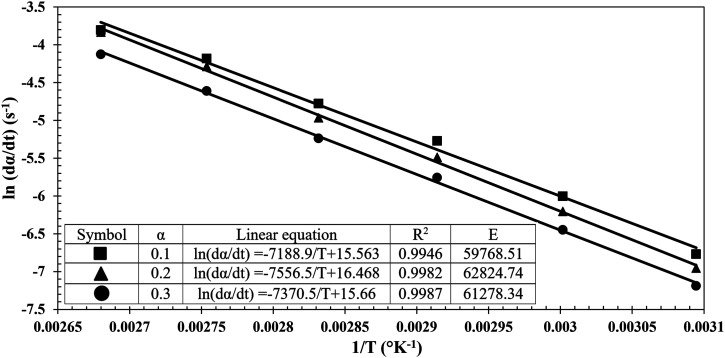
Cure rate as function of inverse absolute temperature at low degree of cure for Gurit Fast Resin System.

The first step is to determination of the activation energy *E*_*a*_ by calculating the slope of ln*(dα/dt)* versus *1/T* at a low degree of cure range (*α* at 0.1, 0.2 and 0.3). An average of 61.29 kJ/mol was found for the activation energy of the Gurit Fast resin system, as seen in Figure 6. The same methodology was implemented for all the resin systems.

The parameters A, m, *n*, were calculated using a least squares nonlinear regression between the cure rate and the degree of cure for all the isothermal temperatures. An R^2^ fitting value is expected to be higher than 0.95 to have a good agreement with the experimental data. equation (4) was used for the Gurit Standard system in order to reach the desired R^2^ fitting value. The other resin system achieved a good agreement with equation (3).

**Figure 7.**
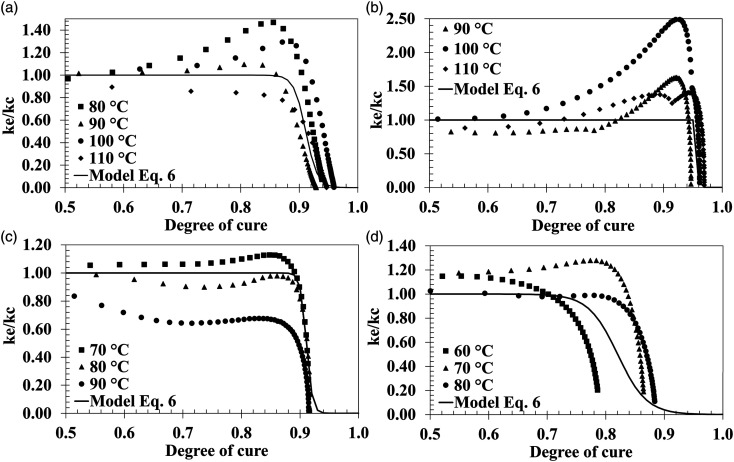
Experimental and cure kinetics model cure rate relationship, $K_{e}/K_{c}$ , as a function of the degree of cure. (a) Hexion. (b) Airstone. (c) Gurit Standard. (d) Gurit Fast.

**Figure 8.**
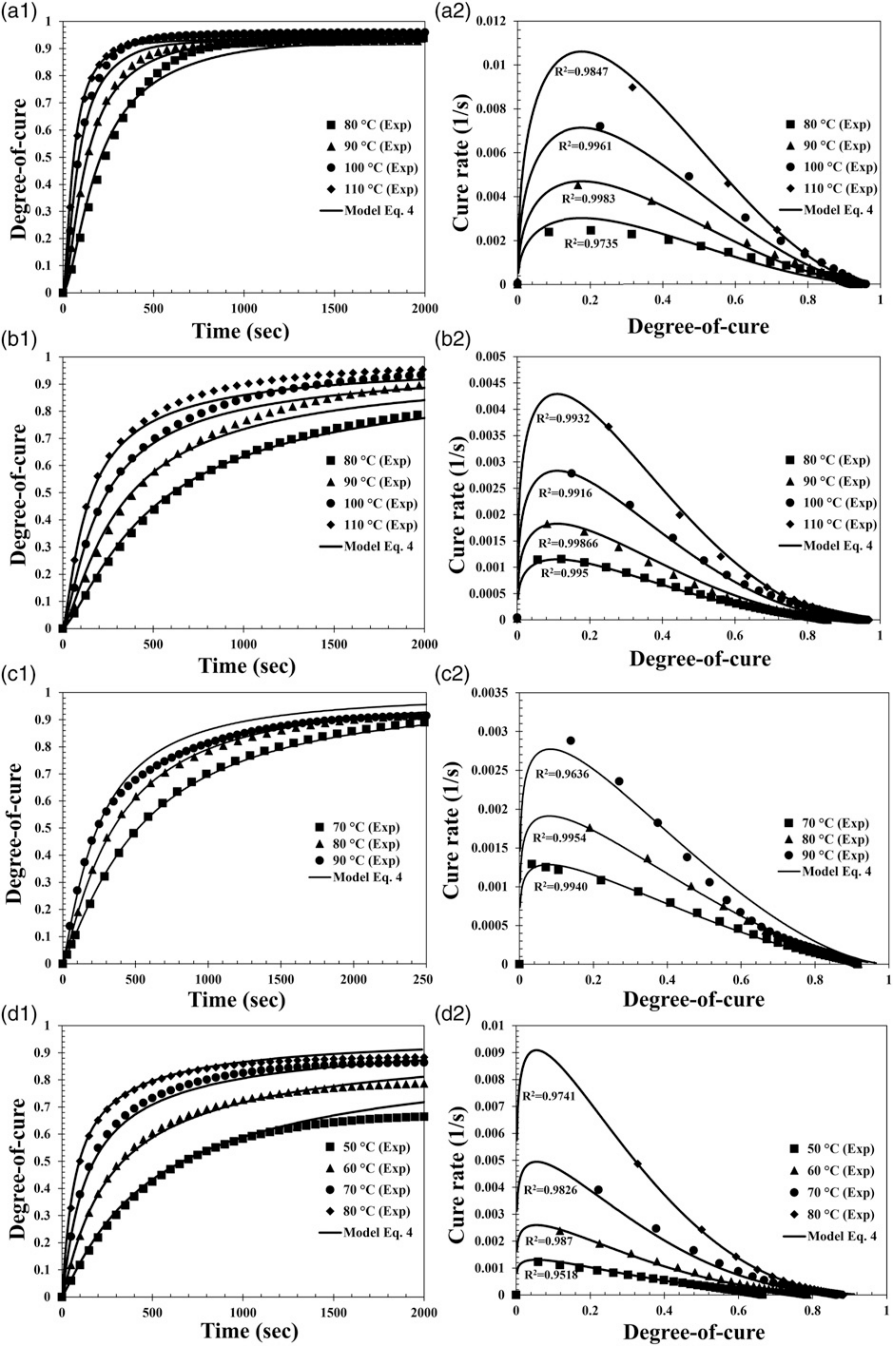
Comparison of experimental data with the cure kinetic model at different isothermal tests. (a) Hexion. (b) Airstone. (c) Gurit Standard. (d) Gurit Fast. (1) Evolution of degree of cure with time. (2) Cure rate as a function of the degree of cure.

A diffusion term can be implemented to improve the cure kinetics model. Cole et al. proposed a model correction with the factor in equation (6). Figure 7 shows the comparison of $K_{e}/K_{c}$ for the different isothermal temperatures and the model of equation (6). There is not a clear dependency of temperature, which makes the term $\alpha_{CT}$ equal to 0. The values of C and $\alpha_{C0}$ are the average of the least squares nonlinear regression of the independent isothermals. Tables 3 and 4 summarize the cure kinetics parameters found on the previous fitting steps. Figure 8 shows the comparison of the experimental data with the cure kinetics model.

**Table 3.** Cure kinetics parameters of the highly reactive thermosets. For temperatures below a critical temperature T_C._

Resin	*Eqn*	*H*_*T*_ (J/g)	*A*_*1*_ (1/s)	*E*_*a1*_ (J/mol)	*A*_*2*_ (1/s)	*E*_*a2*_ (J/mol)	*m*	*n*	T_C_ (°C)
Hexion	3	487	94,100	48,070	—	—	0.34	1.67	110
Airstone	3	410	56,940	49,300	—	—	0.28	2.32	110
Gurit standard	4	410	−53,090	52,160	12,280	43,750	0.10	1.69	90
Gurit fast	3	440	19 × 10^7^	61,290	—	—	0.15	2.63	80

**Table 4.** Diffusion parameters of the highly reactive thermosets.

**Resin**	***C***	**α**_**C0**_	**α**_**CT**_
Hexion	100	0.911	0
Gurit Standard	200	0.911	0
Gurit Fast	40	0.820	0
Airstone	1300	0.958	0

**Figure 9.**
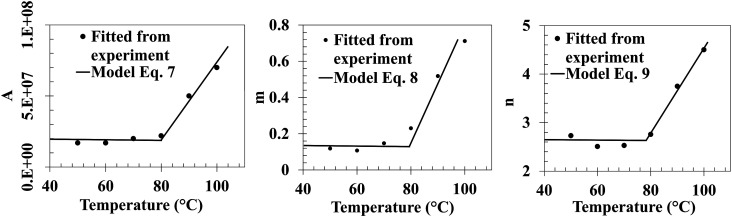
Variation of the parameters A, m and n as a function of the isothermal temperature.

The parameters A, m and n remain constant for temperatures between 50°C–80°C in the case of the cure kinetics model, equation (3), on the Gurit Fast resin system. A linear behaviour of these parameters is observed when increasing the isothermal temperatures starting from a critical temperature $T_{c}$ of 353.15 K (80°C) as seen on Figure 9. The processing temperature range is higher than the critical temperature, thus a logistic based function is implemented to change the constant value of the parameters A, m and n after the critical temperature in a linear relation, Equations (7)–(9).

(7) $$A=A_{0}+\frac{A_{m}T+A_{b}}{1+e^{-\left(T-T_{c}\right)\left(1/K\right)}}$$

(8) $$m=m_{0}+\frac{m_{m}T+m_{b}}{1+e^{-\left(T-T_{c}\right)\left(1/K\right)}}$$

(9) $$n=n_{0}+\frac{n_{m}T+n_{b}}{1+e^{-\left(T-T_{c}\right)\left(1/K\right)}}$$

where *A*, *m* and *n* are function of temperature T in K. *A*_*0*_, *m*_*0*_ and *n*_*0*_ are the values below the critical temperature *T*_*c*_. *A*_*m*_, *m*_*m*_ and *n*_*m*_ are the slope of the linear increment as a function of temperature. *A*_*b*_, *m*_*b*_ and *n*_*b*_ are the intersection of the linear function with *y* axis. Table 5 shows the values found for the parameters of Equations (7)–(9).

**Table 5.** Values of the parameters *A*, *m* and *n* for the Gurit Fast resin system cure kinetics model.

A_0_ (1/s)	A_m_ (1/sK)	A_b_ (1/s)	m_0_	m_m_ (1/K)	m_b_	*n*_0_	n_m_ (1/K)	n_b_
1.9 × 10^7^	2.4 × 10^6^	−8.43 × 10^8^	0.15	0.0241	−8.413	2.63	0.08725	−30.6

## Viscosity modelling

The resin viscosity was modelled using equation (10) following the methods proposed by Khoun et al.^10,21^ Equation (10) takes into account the effects of both temperature and degree of cure. Here, *α*_*gel*_ is the degree of cure at the gel point, and *A*_*µ*_, *E*_*µ*_, *A*, and *B* are constants. The degree of cure was calculated using the cure kinetics equations (3)–(6) from the temperature–time history. The intersection between the storage and loss shear moduli, *G′* and *G″*, was used as a criterion to determine the gel point.^18^

(10) $$\mu =A_{\mu }\left(e^{\frac{E_{\mu }}{RT}}\right){\left(\frac{\alpha_{gel}}{\alpha_{gel}-\alpha }\right)}^{A+B\alpha }$$

Equation (10) can be expressed as a linear relationship between the viscosity and inverse of the temperature

(11) $$\ln\left(\mu \right)=\ln\left(A_{\mu }\right)+\frac{E_{\mu }}{RT}.$$

Linear regression was used to calculate the constants *A*_*µ*_ and *E*_*µ*_ from equation (11), using dynamic data from room temperature until the viscosity starts increasing or viscosity data at the start of the isothermal scan.^22^ The other parameters, *A* and *B* were obtained using a least-squares nonlinear regression between viscosity and temperature with the generalized reduced gradient (GRG) solution method.^23^ Constants calculated for all the resin systems are tabulated in Table 6. The constants were obtained from two experimental dataset for each temperature trial.

**Table 6.** Constant parameters for the viscosity model (Equation (10)) of highly reactive thermoset resins.

Resin	*E*_*µ*_ (kJ/mol)	*A*_*µ*_ (Pa.s)	*α*_*gel*_	*A*	*B*
Hexion	39.64	6.99 × 10^−7^	0.65	−1.96	9.80
Airstone	23.67	2.23 × 10^−5^	0.69	−1.77	5.07
Gurit standard	31.78	1.68 × 10^−6^	0.78	0.80	4.50
Gurit fast	14.79	2.30 × 10^−3^	0.67	1.00	4.40

**Figure 10.**
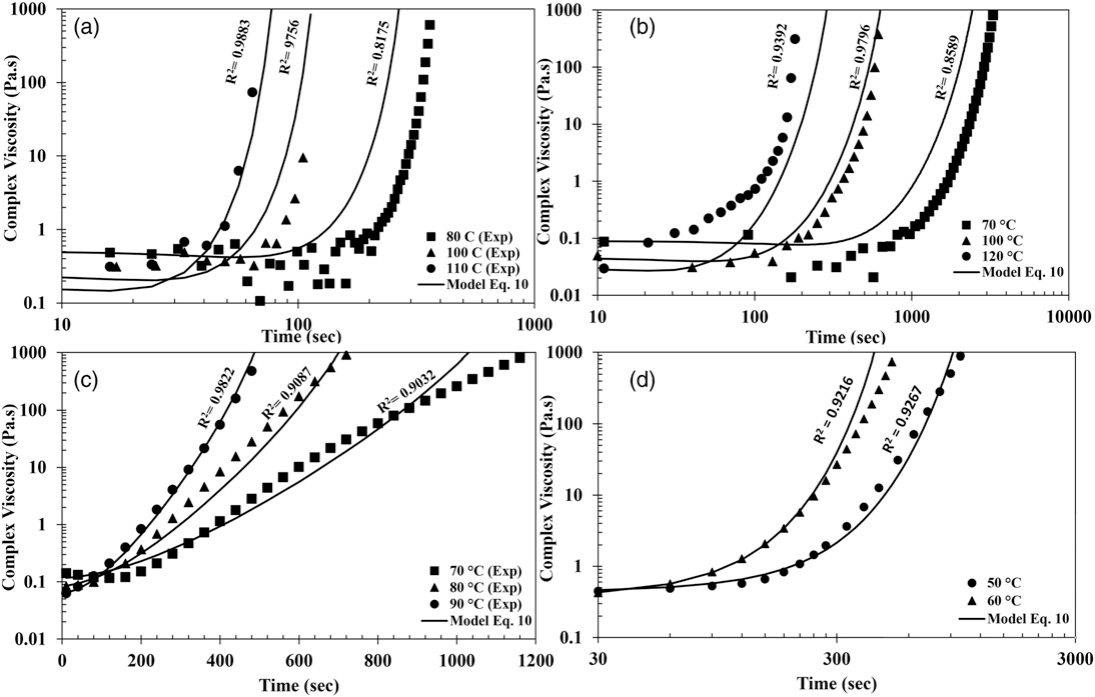
Comparison of experimental data with viscosity model at different isothermal temperatures: (a) Hexion, (b) Airstone, (c) Gurit Standard and, (d) Gurit Fast.

**Figure 11.**
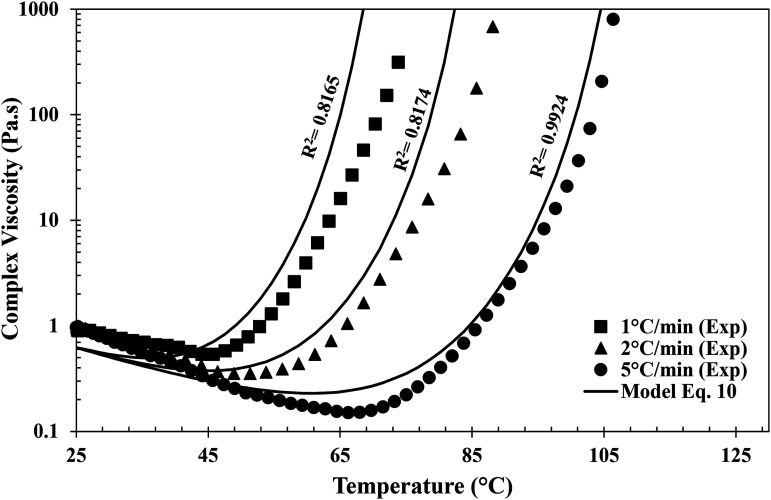
Dynamic experimental data with model fit for Gurit Standard resin.

Figure 10 shows the measured isothermal viscosity and the prediction made by equation (10). The model predicts the evolution of viscosity with an R^2^ value greater than 0.80 for all the resin systems. However, capturing data at high temperatures becomes extremely difficult due to the fast curing nature of the resin systems. The resin would have undergone significant curing reaction by the time the resin is placed between the preheated parallel plates and the sample trimmed to the desired gap. The maximum temperature up to which the isothermals where performed was 120°C. The models developed can also be extended to the dynamic data as shown in Figure 11 for Gurit Standard resin.

## Conclusion and recommendations

Large scale production of composite materials for the transportation industry is mainly constrained by the lack of knowledge on the material processing. CRTM is one of the most suitable processes for an affordable and quick processing of composite materials for large production. Impregnation plays an important role during the processing of composite materials. The overall quality of the final composite part strongly depends on the impregnation of the preform. The resin viscosity plays an important role during impregnation, and the viscosity is directly linked to the cure kinetics evolution of the resin. The work performed on this manuscript focuses on the resin properties and behaviour during CRTM process. The characterization planning consists of 3 stages: I) Thermal stability, II) Cure kinetics, III) and Rheological behaviour.

Arrhenius based cure kinetics models were implemented accounting for diffusion. It was observed that the parameters of these models remain constant below a critical temperature. Isothermal tests were performed above the critical temperature, which shown that the parameters increase linearly. A logistic function was defined to account for this behaviour.

Castro-Macosko model was used to capture the viscosity behavior of all the fast curing resins studied in this work. The model is simple and can be used to predict the processing window necessary for CRTM applications.

**Figure 12.**
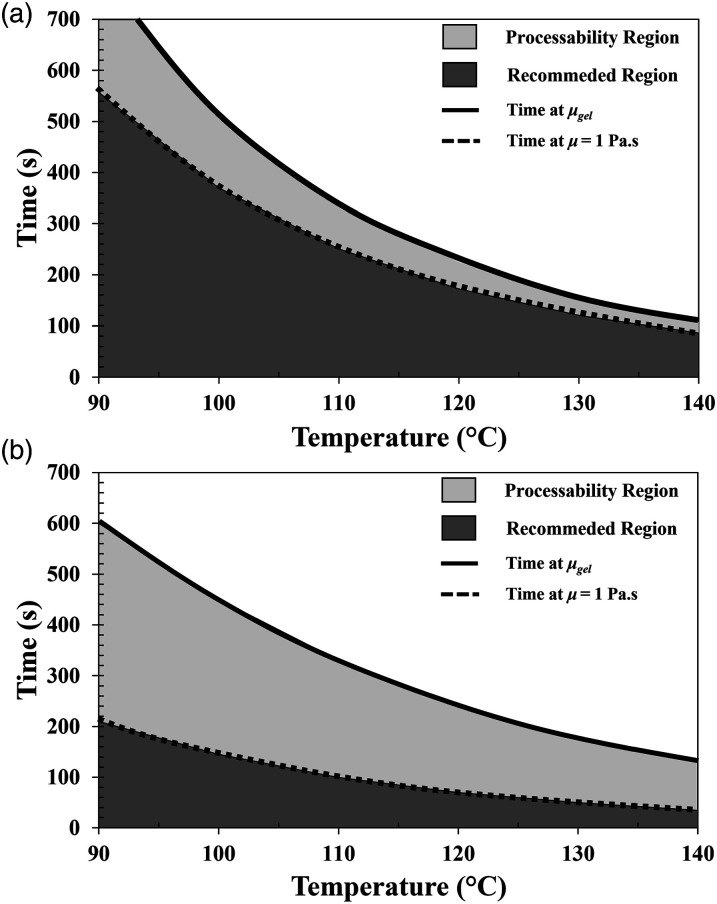
Processability graph defined with the material models for (a) Airstone and (b) Gurit Standard.

The cure kinetics and viscosity models can be used to produce time versus temperature graphs as seen on Figure 12. The time at which the resin reaches the gel point is plotted and used as a limit of processability time. This gel time decreases as the temperature increases from 90°C to 140°C. The region below the gel time curve is named as processability region. The resin can be injected at a specific temperature as long as the injection time does not exceed the gel time. Additionally, a recommended region is defined to ensure a good impregnation of the preform. This recommended region is outlined by the time at which the resin reached a complex viscosity of 1 Pa.s. Figure 12 compares the reactivity of Gurit Standard and Airstone resin systems. Gurit shows a higher reactivity for the same range of temperatures. This means that Gurit can reduce the cure cycle time, but the injection time is very limited to avoid gelation. Airstone on the other hand allows more control of the injection, which is typically required due the complexity and size of some composite parts.

The material models developed here can be implemented in state-of-the-art finite element tools like COMSOL Multiphysics, PAM RTM or COMPRO, RAVEN. These models can be used to solve coupled flow, heat transfer and cure processing problems, to optimize the CRTM process parameters. These models can also be used to solve thermochemical-stress processing problems encountered while making a composite part using CRTM. The detailed procedure and methods explained in this paper can be applied for characterization of wide range of fast curing resin systems.
